# Enhanced forecasting of shipboard electrical power demand using multivariate input and variational mode decomposition with mode selection

**DOI:** 10.1038/s41598-025-06153-z

**Published:** 2025-07-04

**Authors:** Paolo Fazzini, Giuseppe La Tona, Matteo Diez, Maria Carmela Di Piazza

**Affiliations:** 1 Institute of Marine Engineering (INM), National Research Council (CNR), Palermo, Italy; 2 Institute of Marine Engineering (INM), National Research Council (CNR), Rome, Italy

**Keywords:** Energy Management, Shipboard electrical power consumption, Forecasting, Machine Learning, Multivariate Forecasting, Variational Mode Decomposition, Electrical and electronic engineering, Energy grids and networks

## Abstract

Accurate forecasting of shipboard electricity demand is essential for optimizing Energy Management Systems (EMSs), which are crucial for efficient and profitable operation of shipboard power grids. To address this challenge, this paper introduces a novel hybrid forecasting approach that combines multivariate time series decomposition with Machine Learning (ML) techniques. Specifically, the method utilizes Long Short-Term Memory (LSTM) networks to generate forecasts from multivariate input time series that have been decomposed using a newly formulated Variational Mode Decomposition (VMD), termed Variational Mode Decomposition with Mode Selection (VMDMS). VMDMS enables a selective detection process, identifying modes across channels that synergistically enhance forecasting accuracy. The proposed hybrid forecasting method is validated using a dataset of electric power demand time series collected from a real-world large passenger ship. Experimental results confirm the effectiveness of the approach, extending the applicability of VMD to multivariate forecasting without imposing restrictive assumptions on the data. This work contributes to ongoing efforts in optimizing decomposition methods for predictive modeling in energy management, opening new avenues for improving shipboard power grid efficiency.

**Supplementary Information:**

The online version contains supplementary material available at 10.1038/s41598-025-06153-z.

## Introduction

Forecasting high-volatility time series presents one of the most persistent challenges across numerous scientific and engineering domains. From predicting atmospheric phenomena such as wind patterns^1,2^ and short-term wind forecasting^3^ to precipitation events^4^, from temperature fluctuations^5^ to forecasting the complex systems that depend on these variables, inherent uncertainty and chaotic behavior persist as fundamental obstacles in predictive modeling. These forecasting challenges are particularly pronounced in systems characterized by non-linear dynamics, multiple interacting variables, and sensitivity to initial conditions. The difficulty intensifies when considering systems with second-order dependencies on atmospheric variables. In such scenarios, forecasting errors propagate and often amplify through complex causal chains. For instance, predicting electric load demands for critical infrastructure requires not only understanding the direct consumption patterns but also accounting for how atmospheric conditions influence those consumption behaviors. This multi-layered forecasting problem appears across diverse applications, from predicting urban power grid demands during extreme weather events to estimating agricultural irrigation needs during drought conditions. Maritime environments represent a particularly demanding context for such forecasting endeavors. Ships operate at the literal interface of multiple volatile systems, navigating through dynamic ocean conditions while managing complex onboard power systems. The forecasting challenge in this domain is compounded by the constant interaction between vessel operations and atmospheric conditions, where wind resistance, wave patterns, and temperature variations directly impact energy requirements for propulsion, climate control, and other onboard systems.

The shipping industry plays a crucial role in world trade and economy and is responsible for nearly 3% of global greenhouse gas (GHG) emissions^6^. It is also expected that the demand for maritime trade will grow by about 40% over the next three decades^7^. To promote low carbon operations and environmental sustainability in the maritime sector, the International Maritime Organization (IMO) has introduced several mandatory energy efficiency regulations aiming to gradually reduce GHG emissions to net zero by 2050^8^. Among technical solutions proposed to achieve such a decarbonization goal, shipboard energy management systems (EMSs) have emerged as a promising operational measure, with an expected impact on ships’ carbon intensity reduction of up to 10%^9^.

The essential functions of an EMS in a microgrid include monitoring and forecasting of power generation and load demand, assigning generation schedules for energy sources and storage units, managing controllable loads, and handling energy demand according to the set objectives^10^.

Accurate forecasting of electrical load demand, specially multi step-ahead prediction in the day-ahead horizon, is crucial for EMSs used in any electrical microgrid. In addition, in maritime application case, the presence of fluctuating sea conditions, propulsion loads, and possible communication failures can lead to significant discrepancies in forecasted data, thereby complicating the management of shipboard power systems. Therefore, to operate shipboard power systems in effective and efficient way, it is essential to implement a resilient energy management approach that integrates highly performing forecasting methods^11^. Indeed, accurate load forecasting mitigates the effects of uncertainty on load profiles, enabling the EMS to make the most appropriate decisions^12^.

A relevant factor in developing forecasting models is the selection of a suitable prediction horizon. Short-term forecasting has been investigated for its critical role, with distinctions made between extremely short^13^ (up to 6.5 minutes, with 5s granularity) and houry horizons^14^ (4 hours with 15m granularity). In this work, we focus on a forecasting horizon extending up to 8 hours, with a 10m granularity, allowing for high-resolution predictions tailored to maritime power systems. Additionally, we refine this window by analyzing smaller sub-ranges to systematically evaluate the strengths and limitations of the proposed approach.

Electric load forecasting has been widely explored in time series research with methods ranging from statistical approaches to data-driven techniques such as support vector regression, fuzzy systems, and extreme learning machines^15,16^.While statistical models have been extensively used, they require strict assumptions about the data, such as stationarity and non-heteroscedasticity^17^. In contrast, artificial neural networks (ANNs) proved their ability to model complex, nonlinear processes without the above assumptions^18,19^. Deep Learning (DL) methods, particularly, recurrent neural networks (RNNs) and long short-term memory networks (LSTMs), have gained popularity for time series prediction, with LSTMs overcoming issues like vanishing gradients^20^. Time series forecasting methods based on bare LSTM models anyway face several limitations, including accumulated errors, weakening temporal correlations, and a lack of interpretability, which negatively impact prediction accuracy^21^. Moreover, it should be observed that the DL approach is well suited to large datasets and this can pose a challenge when the available dataset is small, as is often the case with electrical power production and consumption data^22,23^.

To enhance forecasting performance, the current trend for variables related to electrical generation and consumption is the use of data-driven Machine Learning (ML) approaches, developed as either hybrid or ensemble forecasting models. Rahimi et al.^24^ demonstrated this with reference to solar power, while Liu et al.^25^ focused on industrial and commercial building power demand. In particular, the use of input data mode decomposition methods, such as Variational Mode Decomposition (VMD)^26^, combined with ML forecasting models, is becoming increasingly popular due to the highly encouraging forecasting results.

VMD^26^ decomposes time series into “well-behaved” sub-signals, called Intrinsic Mode Functions (IMFs) or simply modes. In forecasting applications VMD serves as a preprocessing step to decompose time series into more regular components to facilitate the prediction step. Several authors have applied such an approach to the forecasting of electric power consumption profiles, using univariate data analysis. A VMD-based decomposition step prepended to a Multi-Layer Perceptron (MLP) Artificial Neural Network (ANN) was shown to outperform both a classical autoregressive moving average (ARMA) model and a bare MLP ANN^27^. Zhang et al. prepended a VMD-based decomposition to a Support Vector Regression (SVR) model further combined with a self-recurrent mechanism^28^. The technique proved superior to classical statistical methods and to other ANN and SVR-based methods. VMD-based preprocessing was also combined with deep learning (DL) architectures like a deep bidirectional Long Short-Term Memory (LSTM) network with an attention mechanism^29^ and a temporal convolution network followed by an LSTM with self attention^30^. In particular, Xiong et al. combined the modes resulting from VMD with other external features like temperature and seasonality^30^. Finally, Wang et al. enhanced the accuracy of DL-based load forecasting by modelling the impact of multi-dimensional meteorological inputs on electrical load power and by applying VMD to data^31^. In this approach, VMD parameters such as the balancing parameter of the data-fidelity constraint and the number of IMFs generated are determined by the marine predators algorithm (MPA) and the forecasting task is performed by cascading an LSTM-based ANN architecture. It should be observed that none of these approaches considered shipboard electric power consumption.

While VMD has been employed as a preprocessing step for mode-level forecasting, it is constrained to analyzing a single time series input. Critically, VMD lacks effective criteria for enabling cooperative mode-level forecasting that could leverage multivariate time series data for prediction. MVMD (^32^) addresses this limitation by establishing alignment between mode central frequencies. This frequency alignment provides the essential criterion for managing modes derived from different channels and opens a new interesting perspective since multivariate analysis is able to exploit complex relationships in the data by revealing patterns that may be overlooked when considering variables separately. To the best of authors’ knowledge such an approach has not been exploited for electrical load demand forecasting. However, it was applied to other time series forecasting approaches. In particular, Wang et al. proposed a forecasting method for the Air Quality Index^33^. Multivariate VMD was utilized to decompose a multivariate time series consisting of ternary interval-valued time series of the air quality index and meteorological variables. Subsequently, a multivariate relevance vector machine model was employed to individually forecast each of the resulting multivariate modes. Similarly, multivariate VMD was also used as a preprocessing step for an interval-valued wind speed forecasting^34^ and for ocean wave height forecasting^35^. Notably, in both cases, the variables constituting the time series are homogeneous or at least exhibit similar behaviors.

The MVMD extension of VMD to multivariate time series^32^ imposes the same central frequency among components of a multivariate input. Such a requirement may not be applicable to forecasting tasks when the components of multivariate data are heterogeneous variables or, while homogeneous, they do not share frequency spectrum similarities. Specifically, depending on the dataset size and the number of decomposition modes, the procedure may introduce inaccuracies that uniformly affect all time series channels. This becomes particularly problematic when the target time series of the forecast is significantly impacted, as MVMD offers no mechanism to preserve the integrity of this specific time series, and decomposition errors can substantially degrade the overall forecasting performance.

Some authors proposed methods to overcome this limitation. Zheng et al. presented a method for forecasting of evapotranspiration from multivariate input with heterogeneous variables^36^; here, a multivariate VMD was applied, followed by a soft feature filter to appropriately weight each mode. This ensured that no significant information was lost due to the heterogeneity of the input variables. The resulting modes were then used as input to a recurrent ANN. Finally, another approach for forecasting natural gas prices from multivariate time series input with heterogeneous variables proposed to combine multivariate empirical mode decomposition as a primary decomposition followed by multivariate VMD as secondary decomposition^37^. However, the cited approaches significantly increase the overall complexity of the forecasting procedure.

In our work a novel approach for shipboard electrical power demand forecasting is developed which relies on a unique integration of VMD with ML-based forecasting from multivariate input. Such an approach allows for enhancing the accuracy of the power demand predictions without implying increased complexity of the forecasting procedure.

Our proposed methodology circumvents the issues of MVMD by providing an alternative approach: rather than imposing frequency-alignment constraints, it implements a posterior selection of modes that can operate cooperatively, based on the proximity of their central frequencies. The quantification of this proximity is determined through a procedure that establishes a narrow acceptance region in the low-frequency range that progressively widens toward higher frequencies. A tunable parameter allows users to calibrate the mode selection process to their specific requirements. Notably, this procedure defaults to standard VMD when the generated frequencies are excessively disparate.

Specifically, a novel formulation of VMD, called Variational Mode Decomposition with Mode Selection (VMDMS) is designed to integrate decomposition with the task of time series forecasting from multivariate input which is performed by ANNs based on a Long Short-Term Memory (LSTM) model. The proposed hybrid forecasting method, i.e. VMDMS-LSTM, relies on the following features:

1. VMDMS decomposes the multivariate input time series using univariate VMD, then recombines potential cooperating modes in an innovative manner;
2. VMDMS builds naturally on the univariate power forecasting method, which has proven to be successful in^38^;
3. VMDMS is simple and fast and it does not impose to the decomposition procedure that the variables of the input time series share the same central frequency;
4. VMDMS-LSTM is tested on a sample of electric power demand time series (not having frequency spectrum similarities) collected on a real-world large passenger ship.

The use of VMDMS-LSTM results in an improvement with respect to the method based only on univariate VMD and LSTM, thus highlighting the potential of hybrid methods based on decomposition and machine learning in the context of forecasting of electrical power data from multivariate time series input. The source code for the proposed method is available at https://github.com/pfaz69/VMDMS/tree/main.

The remainder of this work is organized as follows: first, the proposed method is described in detail, including the novel VMD formulation and the followed partitioning strategy; then, a description of the case study and the analysis of the setup are provided; subsequently, the decomposition and forecasting results along with a discussion are presented; finally, conclusions are drawn in the last section.

## The proposed method

**Fig. 1:**
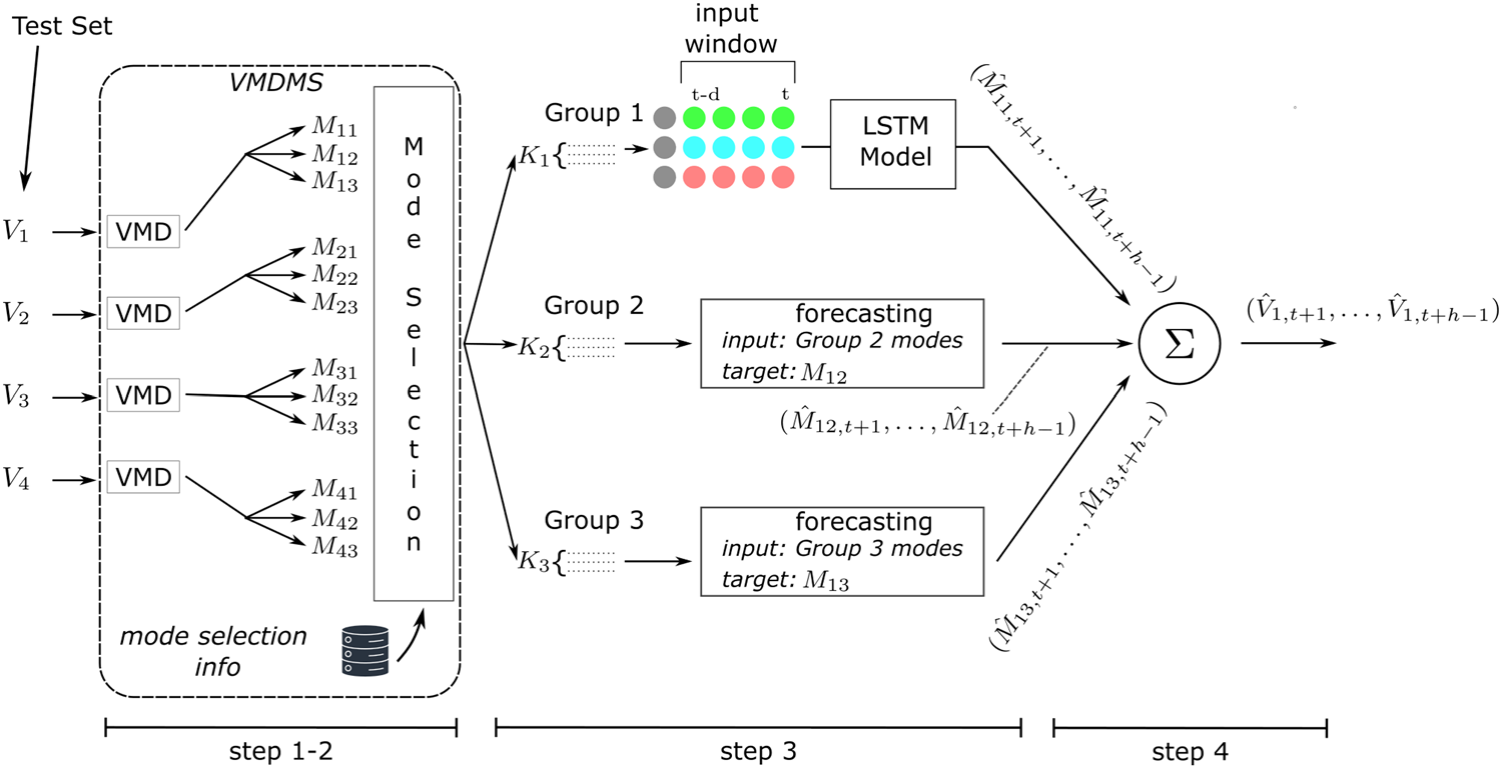
General scheme of the proposed method.

**Fig. 2:**
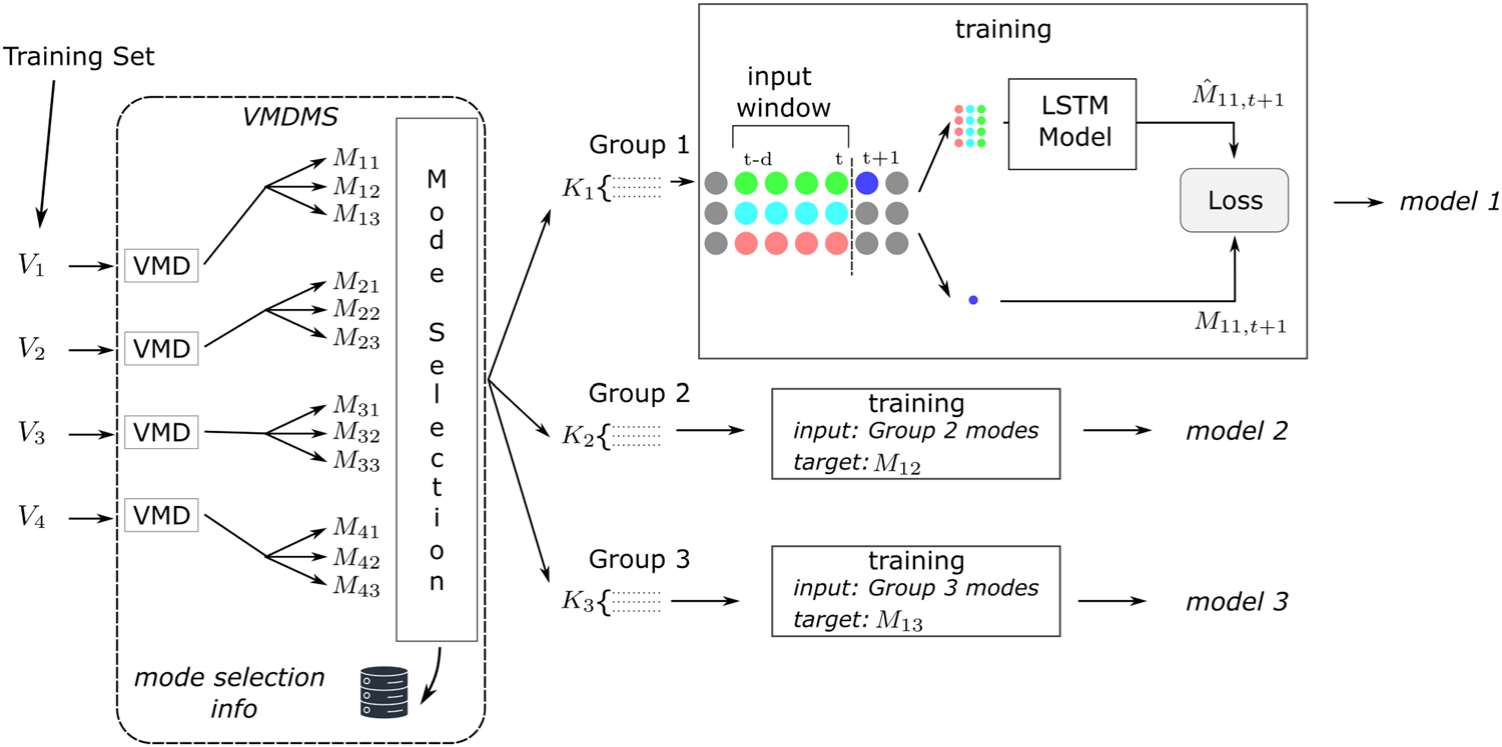
Training of the models for the proposed method.

### Nomenclature

The relevant nomenclature used in this work is summarized in this sub-section, for the sake of clarity.

A time series is a set of data points indexed by time, usually taken at regular time intervals. Formally it is defined as:

1 $$\begin{aligned} \{x_i\}_{i=1}^T, x_i \in \mathbb {R}^N \end{aligned}$$

where *i* is the time step index, *T* is the length of the time series, and *N* is the number of variables composing the time series. If $N=1$ the time series is univariate, otherwise it is multivariate. Electrical power demand time series consist of active power demand measurements of one or more electrical loads.

Forecasting is the task of predicting the next value of the time series $x_{i+1}$ given the previous observations $(x_i,\ldots ,x_1)$. Furthermore, it is usually desired to forecast the series for a horizon *h* in the future $(x_{i+h},\ldots ,y_{i+1} )$, such a task is called multi-step forecasting.

The task of forecasting only one of the variables of a multivariate time series is referred to as ’$N-1$ forecasting’ or also forecasting from multivariate input and the forecasted variable is referred to as target variable. Conversely, the task of forecasting a univariate time series (without considering additional variables) is called in this paper ’$1-1$ forecasting’ and it is used as a benchmark method.

The Intrinsic Mode Functions (IMFs) resulting from VMD decomposition will also be referred to as sub-signals or simply as modes.

### The algorithm

The proposed method involves four main steps:

1. *Input Time Series Decomposition by VMD*: following literature in forecasting with VMD preprocessing^28–31^ the $N$ variables of the multivariate time series used as input are individually divided into sub-signals, each confined to specific frequency ranges centered around a central frequency.

2. *Mode Partitioning*: the sub-signals are then grouped in a manner that allows them to contribute synergistically to the subsequent training and prediction stages. Sub-signals with similar central frequencies are grouped together, while those corresponding to isolated frequencies are discarded. The number of groups matches the number of sub-signals into which each component of the multivariate input time series is decomposed. The machinery adopted to form these groups represents the main novelty introduced in this paper, never proposed before, to the best of the authors’ knowledge. The ensemble of steps 1 and 2 constitute the newly proposed VMD method, i.e., the VMDMS.

3. *ANN-based Forecasting*: each mode group undergoes a prediction process utilizing an ANN architecture based on Long Short-Term Memory (LSTM) cells. Each ANN is trained using as input the modes composing the corresponding group and as output the corresponding mode of the target variable. In the forecasting execution, the result of this step is the prediction of the modes of the target variable.

4. *Forecasting Recombination*: the predicted modes are finally summed up together to form the complete forecasting of the target variable.

Figure 1 illustrates a streamlined scenario related to a sample case where the forecasting of the target variable $V_1$ is obtained from a multivariate time series input, composed of four variables ($V_1$, $V_2$, $V_3$ and $V_4$). In this example, first, $V_1$, $V_2$, $V_3$ and $V_4$ are each decomposed into 3 distinct modes. These modes are then grouped or discarded by the “Mode Selection” block depending on how close the respective central frequencies are with respect to the central frequencies of the modes of the target variable $V_1$. Following this, each group is utilized as an input to predict the future values of the corresponding mode of the target variable (”Forecasting” blocks). The final step of the process involves adding up these predicted modes to form an overall prediction for the target variable of the time series.

The details of LSTM models’ training is illustrated in Figure 2.

The following subsections will provide a comprehensive explanation of each step involved in the process.

### Variational mode decomposition

VMD^26^, is a signal processing approach that decomposes a univariate time series into a collection of Intrinsic Mode Functions (IMFs), simply referred as *modes*. This method was developed as an advancement over other decomposition techniques such as Empirical Mode Decomposition (EMD)^39^. In the proposed approach, VMD is used to individually decompose each of the variables of the input multivariate time series.

In VMD, each mode represents a distinct frequency band of the original time series. The design of this decomposition ensures that the sum of all the modes recreates the original time series with minimal errors. A defining feature of this method is the significantly slower variation of both amplitude and frequency compared to the phase, with their frequency content strategically positioned around specific points along the original spectrum called central frequencies.

VMD operates on a variational formulation, where the algorithm iteratively solves an optimization problem via Alternating Direction Method of Multipliers (ADMM)^40^ to identify the central frequencies and the modes that minimize the bandwidths.

Formally the above described optimization problem takes the form of constraint minimization:

2 $$\begin{aligned} & \min _{\left\{ u_k\right\} ,\left\{ \omega _k\right\} }\sum _k\left\| \partial _t\left[ \left( \delta (t)+ j/(\pi t)\right) * u_k(t)\right] exp(-j \omega _k t)\right\| _2^2 \end{aligned}$$

3 $$\begin{aligned} & \text{ s.t. } \quad \sum _k u_k=f \end{aligned}$$

Equation (2) articulates the variational principle that needs to be minimized by identifying the appropriate modes ($u_k$) and shifting (central) frequencies ($\omega _k$) where the index *k* identifies the specific mode. Notably:

- *f* is the time series to reconstruct;
- $\delta (t)$ is the Dirac Delta Distribution;
- $j/(\pi t)$ is the Hilbert transform impulse response;
- $\left[ \left( \delta (t)+ j/(\pi t)\right) * u_k(t)\right]$ is the analytic signal corresponding to the mode $u_k(t)$;
- $exp(-j \omega _k t)$ is the shift towards the central frequency of each mode $u_k(t)$.

Equation (3) defines the reconstruction constraint, where the summation runs over all the modes. Equation (2) and (3) can be reformulated to the following variational principle:

4 $$\begin{aligned} \begin{aligned} \mathscr {L}\left( \left\{ u_k\right\} ,\left\{ \omega _k\right\} , \lambda \right) := \alpha \sum _k\left\| \partial _t\left[ \left( \delta (t)+j/(\pi t)\right) * u_k(t)\right] exp(-j \omega _k t)\right\| _2^2 \quad +\left\| f(t)-\sum _k u_k(t)\right\| _2^2 + \left\langle \lambda (t), f(t)-\sum _k u_k(t)\right\rangle \end{aligned} \end{aligned}$$

where $\alpha$ is a weighing parameter and $\lambda (t)$ is the parameter of the Lagrangian multipliers term enforcing the norm constraint. Making use of the Parseval/Plancherel Fourier isometry under the norm, this problem can be solved in spectral domain as detailed in appendix.

The solution takes the form of an iterative Wiener filtering of the current residual:

5 $$\begin{aligned} \hat{u}_k^{n+1}(\omega )=\frac{\hat{f}(\omega )-\sum _{i \ne k} \hat{u}_i(\omega )+\hat{\lambda }\left( \omega \right) /2}{1+2 \alpha \left( \omega -\omega _k\right) ^2} \end{aligned}$$

The mode in time domain is obtained as the real part of the inverse Fourier transform of this filtered analytic signal.

The central frequency problem can also be solved iteratively:

6 $$\begin{aligned} \omega _k^{n+1}=\frac{\int _0^{\infty } \omega \left| \hat{u}_k(\omega )\right| ^2 d \omega }{\int _0^{\infty }\left| \hat{u}_k(\omega )\right| ^2 d \omega } \end{aligned}$$

Table 1 shows the practical implementation of the algorithm, via ADMM. Further development details of the VMD are provided in the Appendix.

**Table 1:**
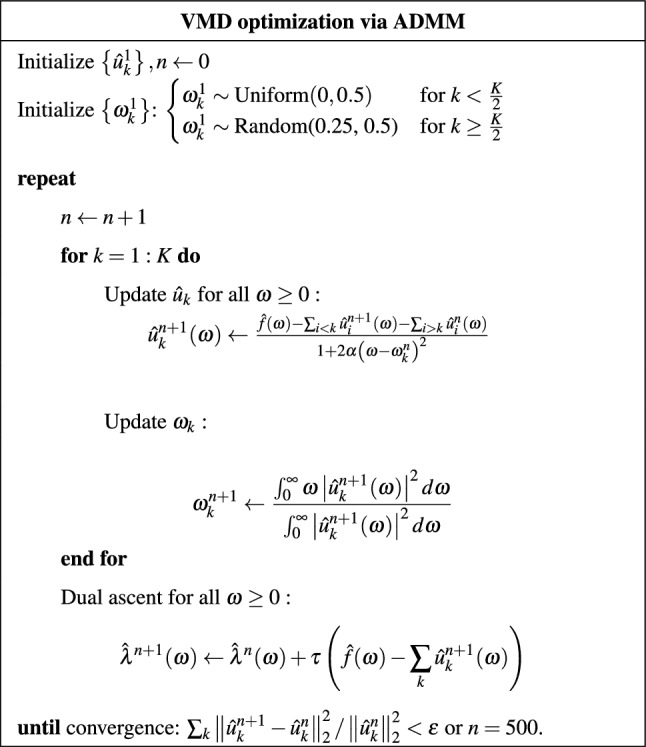
VMD Algorithm Pseudo-Code.

### The partitioning machinery

The mode partitioning step groups the modes resulting from the individually decomposed variables of the input. Modes with similar central frequencies are grouped together, whereas those corresponding to isolated central frequencies are discarded.

The partitioning machinery is implemented according to the algorithm described by the pseudocode shown in Table 2.

Within the partition algorithm, $\tilde{\omega _k}$, represents the vector of central frequencies of the modes of the target variable; $\hat{u_i}$, $\Delta _i$, and $\omega _i$ represent, respectively, the vector of all modes of all variables, the related vector of bandwidths, and the related vector of central frequencies; $\gamma$ is a customizable parameter.

The variable $\tilde{\omega _k}$ iterates over the K modes into which the target variable has been decomposed. At each iteration the algorithm compares the central frequency of the k-th mode ($\tilde{\omega _k}$) with the central frequencies of all the $S = K*N$ modes of all the variables. If the distance $\delta$ between such central frequencies is below a threshold $\gamma *\Delta _k$, the mode $\hat{u_i}$ is added to the k-th group.

**Table 2:**
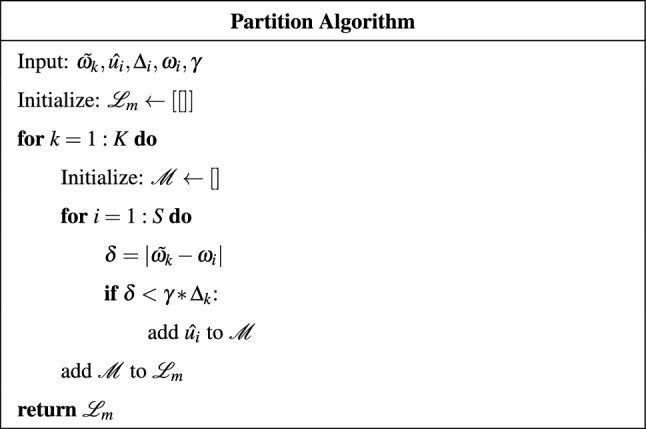
Partition Algorithm.

**Fig. 3:**
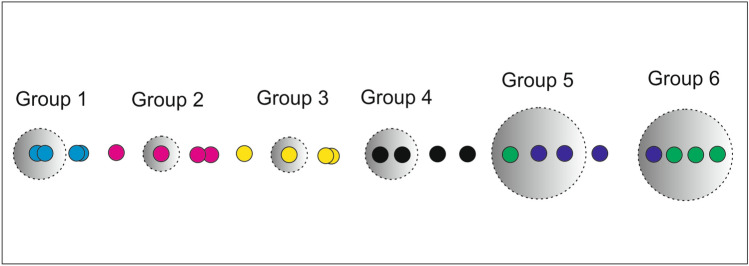
Example of partitioning in the range of frequencies with 6 modes and 4 variables.

Figure 3 illustrates an example of partitioning referred to the case of a multivariate time series composed of 4 variables each of which is decomposed into 6 modes. Different colors of the dots refers to different modes. The grey sets selectively include the relevant modes and excludes the others.

By examining Figure 3, we can see that the groups formed using the proposed partitioning strategy may include modes of the same order (represented by dots of the same color), which result from the decomposition of different variables (as seen in the first four groups from left to right). Additionally, the groups may contain modes of different orders (as observed in the last two groups).

### ANN-based Forecasting and Recombination

The forecasting task is carried out by ANNs equipped with LSTM cells^41^. These cells capture long-term dependencies within the input data sequences and pass the information to a dense layer, each with a single output unit. The dimensions of the input of the ANNs are the number of input time steps and the number of input variables.

Figure 5 shows the overall structure of the ANNs and the detailed structure of LSTM cells. Each LSTM cell comprises a memory unit and three key gates: the input gate, output gate, and forget gate. This design allows LSTM to capture long-term dependencies by controlling which information is ”retained” and which is ”discarded,” enabling a regulated flow of information. Specifically, the input gate updates the cell’s content, while the forget gate determines what information in the memory state should be kept or discarded at any given moment. At the same time, the output gate manages the information that is passed from the memory unit. Rectangular boxes represent activation functions applied after matrix multiplication, while circular spots indicate element-wise operations. In Figure 5, *x*(*t*), *h*(*t*) , and *c*(*t*) correspond to the input, hidden state, and cell activation vector, respectively, with $\sigma$ denoting the sigmoid activation function. The cell’s output is a weighted sum of *x*(*t*), $h(t-1)$, and a bias term, passed through a sigmoid activation.

Multi-step ahead forecasting is performed by iteratively applying an auto-regressive process over the forecasting horizon.

As shown in Figure 1, the method employs the same number of ANNs as the groups produced by the previous step (VMDMS). Each ANN receives as input the corresponding group and gives as output the corresponding mode of the target variable.

The final step of the method, i.e., the recombination of forecast modes, consists of a simple summation of the ANNs’ outputs and provides the forecasted values of the target variable of the multivariate time series.

## Case study and analysis setup

The dataset used in this study is a multivariate time series of shipboard electrical power consumption. Such operational data were collected from a real-world large passenger ship, whose electrical power system is realized according to the Integrated Power System (IPS) concept^42^. In shipboard IPSs a centralized diesel-electric power plant caters to all the power needs on board, including both service and propulsion power^43^.

**Fig. 4:**
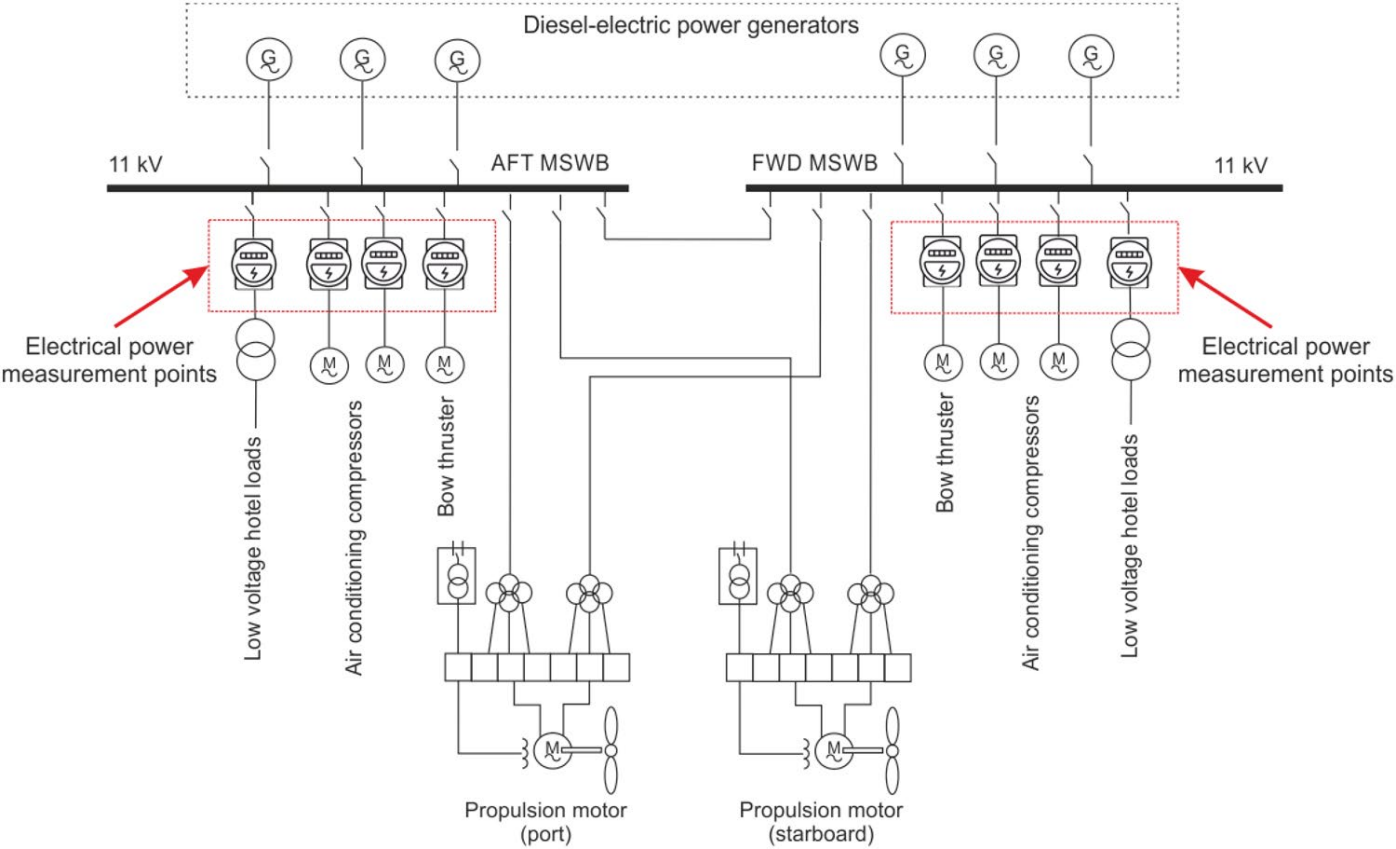
Electrical power system.

The electrical power system of the case study ship includes an 11 kV AC primary distribution system that powers the air conditioning, propulsion, and bow thruster loads. A 690/220 V AC secondary distribution system is also present, providing for supplying power to accommodation loads, galleys, and other auxiliary services, as depicted in Figure 4.

Power measurements were carried out at appropriate connectors of the ship’s high voltage main switchboards, as highlighted in Figure 4.

Data were collected in a period of about four months with a sampling time of ten minutes. Each variable of the available dataset corresponds to the electrical power usage of a different shipboard electrical load.

To evaluate the approach described in the Proposed Method section, we have chosen four variables, denoted as $P_1$, $P_2$, $P_3$, and $P_4$, from the electrical power load time series. Each variable contains 17569 data points that, prior to processing has been scaled in the interval $\left[ -0.5, 0.5\right]$. Considering that each datum is collected every 10 minutes, the total time span is roughly 122 days. The tuning of the ANN hyperparameters is detailed in the validation procedure described in^38^. This paper adopts these parameters without further adjustments.

Four test scenarios were considered; in each scenario one of the four variables was considered as target variable and forecasted using the proposed approach.

**Fig. 5:**
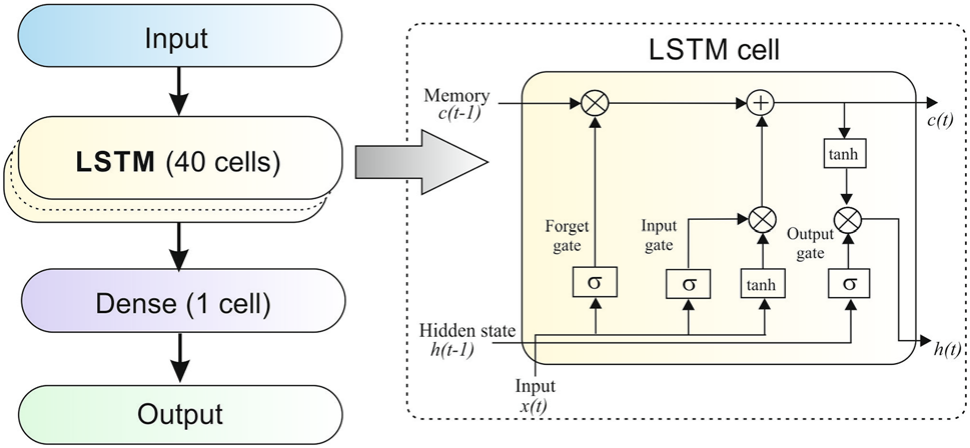
Overall structure of the ANNs and the detaied structure of LSTM cells.

The exploited ANN, whose overall structure is illustrated in Figure 5, is trained using the data from the initial 121 days and the last day is divided into three consecutive 8-hour periods, known as time slot 1, time slot 2, and time slot 3, for validation and testing purposes.

For each scenario, an LSTM model with univariate VMD preprocessing performing 1-1 forecasting (i.e. using the past values of a single time series to forecast its future values) was used as benchmark. The same train, validation, and test sets and the same hyperparameters were employed.

The number of modes *K* was set to 12 for the tests. Such a value was chosen empirically considering that it proved to be a good compromise between reconstruction accuracy and complexity. The 12 central frequencies were initialized as follows: 6 have been uniformly spaced in the frequency interval $\left[ 0, 0.5\right]$; the remaining 6 have been randomly positioned in the interval $\left[ 0.25, 0.5\right]$. Moreover $\alpha$ was set to 2000, $\tau$ to 1 and $\epsilon$ to $exp(-7)$. The maximum number of iterations of the ADMM was set to 500.

Table 3 shows the hyperparameters adopted in the ANN design, based on previous work settings^38^.

**Table 3:** Model hyperparameters.

Hyperparameter	*Value*
*Input Steps for each mode*	*150 140 130 120 100 80*
	*60 40 30 25 20 20*
*Num. of LSTM cells*	*40*
*Epochs*	*5*
*Early Stopping*	*yes*
*Dense Layer Activation*	*tanh()*
*Loss*	0.5*[Mean Squared Error] +
	0.5*[Mean Cross Entropy]

## Results

In this section, we first present the decomposition results of the analyzed time series. Subsequently, we display the results obtained for the 8-hour-ahead forecasting case study, as described in the section *Case Study and Analysis Setup*. We also provide an analysis of error propagation in recursive forecasting, which identifies a forecasting horizon within which the error propagation remains below a specified threshold. Finally, the forecasting results corresponding to this horizon are presented.

### Decomposition results

The decomposition by VMD of the four electrical power time series variables collected on the case study ship ($P_1$, $P_2$, $P_3$, and $P_4$) was performed under the conditions delineated in the *Case Study and Analysis Setup* section.

**Fig. 6:**
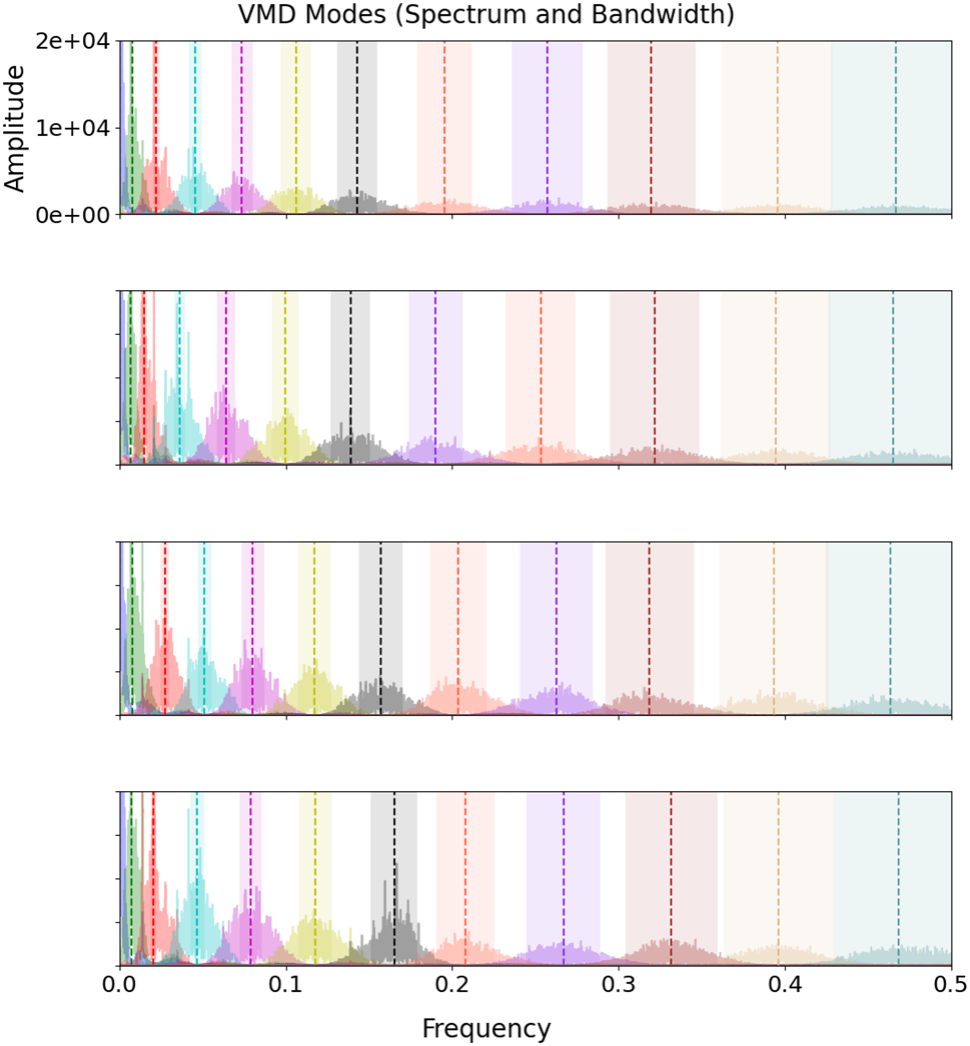
VMD Modes (Spectrum and Bandwidth) in the frequency range [0, 0.5] for $P_1$, $P_2$, $P_3$ and $P_4$.

**Fig. 7:**
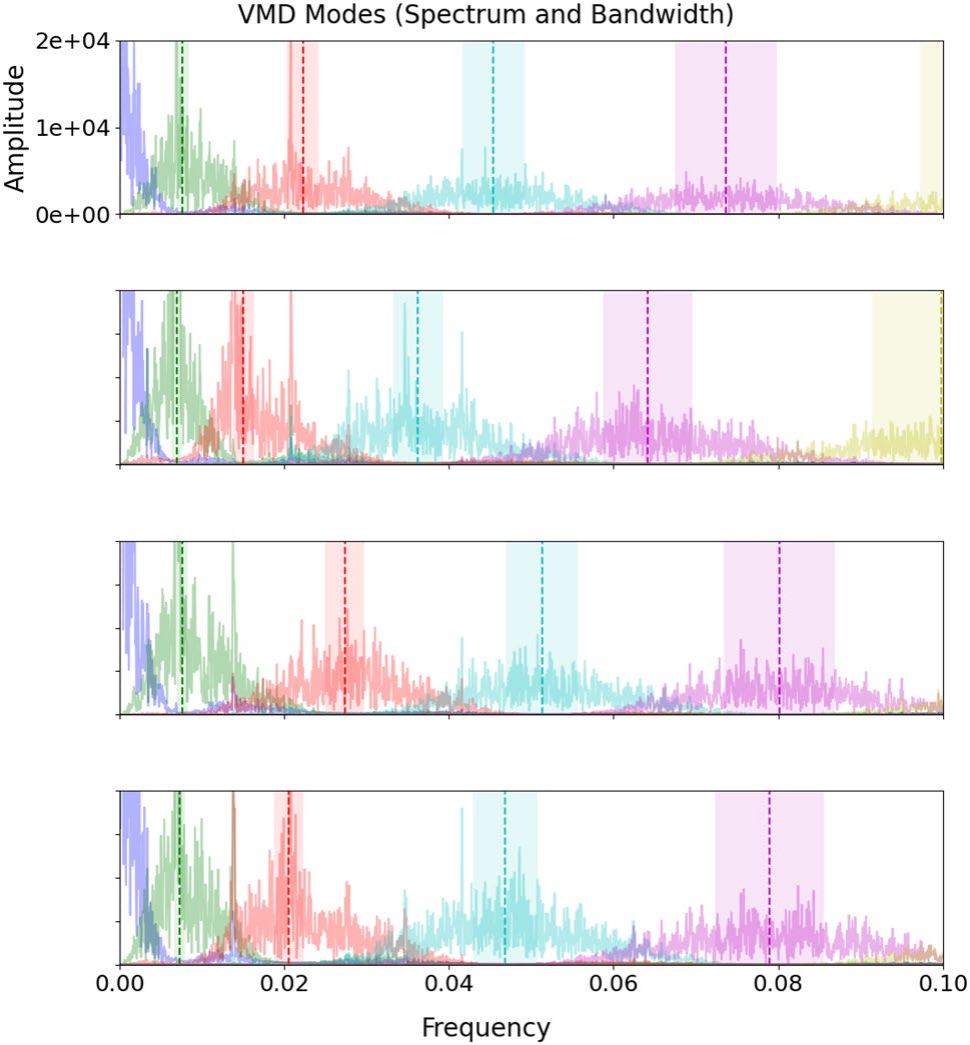
VMD Modes (Spectrum and Bandwidth) in the frequency range [0, 0.1] for $P_1$, $P_2$, $P_3$ and $P_4$.

Figures 6 and 7 show a set of graphs where the $K = 12$ modes generated for each of the four variables under consideration are represented in frequency domain. From these graphs it is observed that modes of different orders from different variables tend to have regions of overlap and that they may have closer central frequencies than modes of homologous order. This result consolidates the choice of grouping the modes obtained by processing the multivariate input time series through VMD according to the partitioning method described above.

In addition, tables 4, 5, 6 and 7 illustrate the participation of each mode in each of the four target variable forecasting. It is observed that multiple unnecessary modes are discarded by partitioning, implying that the neural network will be able to process ‘simpler’ inputs but still containing adequate information to obtain a well-accurate prediction results.

**Table 4:** Modes exploited to forecast $P_1$.

	$M_{1}$	$M_{2}$	$M_{3}$	$M_{4}$	$M_{5}$	$M_{6}$	$M_{7}$	$M_{8}$	$M_{9}$	$M_{10}$	$M_{11}$	$M_{12}$
$P_1$	1	2	3	4	5	6	7	8	9	10	11	12
$P_2$	1						7	9	8	10	11	12
$P_3$	1	2						8	9	10	11	12
$P_4$	1	2		4					9	10	11	12

**Table 5:** Modes exploited to forecast $P_2$.

	$M_{1}$	$M_{2}$	$M_{3}$	$M_{4}$	$M_{5}$	$M_{6}$	$M_{7}$	$M_{8}$	$M_{9}$	$M_{10}$	$M_{11}$	$M_{12}$
$P_1$	1						7	9	8	10	11	12
$P_2$	1	2	3	4	5	6	7	8	9	10	11	12
$P_3$	1							9		10	11	12
$P_4$	1									10	11	12

**Table 6:** Modes exploited to forecast $P_3$.

	$M_{1}$	$M_{2}$	$M_{3}$	$M_{4}$	$M_{5}$	$M_{6}$	$M_{7}$	$M_{8}$	$M_{9}$	$M_{10}$	$M_{11}$	$M_{12}$
$P_1$	1	2						8	9	10	11	12
$P_2$	1								8	10	11	12
$P_3$	1	2	3	4	5	6	7	8	9	10	11	12
$P_4$	1	2			5	6	7	8	9	10	11	12

**Table 7:** Modes exploited to forecast $P_4$.

	$M_{1}$	$M_{2}$	$M_{3}$	$M_{4}$	$M_{5}$	$M_{6}$	$M_{7}$	$M_{8}$	$M_{9}$	$M_{10}$	$M_{11}$	$M_{12}$
$P_1$	1	2		4					9	10	11	12
$P_2$	1									10	11	12
$P_3$	1	2			5	6		8	9	10	11	12
$P_4$	1	2	3	4	5	6	7	8	9	10	11	12

### Forecasting results

**Fig. 8:**
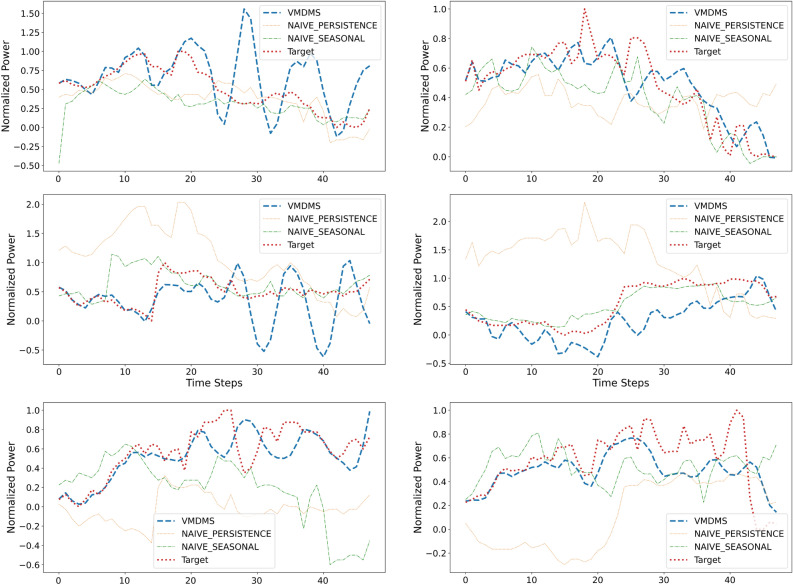
Forecasting results for the normalized power values, $P_1$ and $P_2$, over an 8-hour time window, compared against two baseline methods: Persistence, which repeats the past 8 hours of observations, and Seasonal Persistence, which repeats the past 8 hours of observations taken from the same time period in the previous 24-hour cycle. First column: Forecasts for the time slots 1, 2, and 3 of $P_1$ (top to bottom). Second column: Forecasts for the time slots 1, 2, and 3 of $P_2$ (top to bottom). In the graphs, the orange line represents observation (Target), while the blue line depicts the results of the VMDMS multivariate forecasting. The graphs have been normalized by dividing by the maximum observed values of ($P_1$) and ($P_1$) over the 24-hour test period.

The accuracy of the proposed forecasting method was evaluated under the conditions delineated in the *Case Study and Analysis Setup* section.

#### Results within the 8-hour-ahead time horizon

Multi-step ahead forecasting of powers $P_1$ and $P_2$, $P_3$, and $P_4$ was performed considering an 8-hour-ahead time horizon. Such a forecasting horizon corresponds to 48-time steps-ahead, considering that the time series sampling time equals to 10 minutes.

**Fig. 9:**
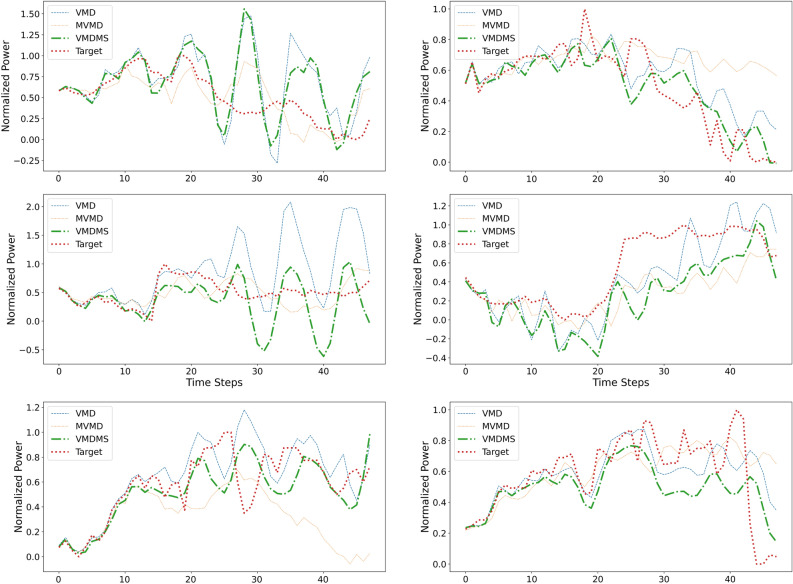
Forecasting results for the normalized Powers $P_1$ and $P_2$. First column: Forecasts of VMD, MVMD and VMDMS for the time slots 1, 2, and 3 of $P_1$ (top to bottom). Second column: Forecasts for the time slots 1, 2, and 3 of $P_2$ (top to bottom). The graphs have been normalized by dividing by the maximum observed values of $P_1$ and $P_2$ over the 24-hour test period.

Time-domain forecasting results for the powers $P_1$ and $P_2$ are presented in Figure 8, along with two popular alternatives, namely Persistence and Seasonal Persistence. The evaluation time window consists in 24 hours subdivided in the three 8-hour time slots (time slots 1, 2, and 3) defined above. Powers represented in Figure 8 are suitably normalized by dividing by the maximum observed values of $P_1$ and $P_2$ over the 24-hour test period to respect operational data confidentiality. In each graph, the orange line is the true target variable and the blue line is the result of the forecasting from multivariate input with VMDMS (N-1). The graphs in Figure 8 allow to qualitatively appreciate the VMDMS forecasting accuracy: the algorithm performs remarkably well in the first quarter of each slot and then its effectiveness decreases over time. Similarly, Figure 9 presents a graphical comparison of the results obtained with the proposed method against those achieved using VMD (1-1) and MVMD.

To obtain a quantitative assessment of the proposed method’s accuracy, also in comparison with MVMD^32^ and the univariate forecasting (1-1), Normalized Root Mean Squared Error (NRMSE), Normalized Mean Absolute Error (NMAE) and Symmetric Mean Absolute Percentage Error (sMAPE) have been evaluated in all the considered test scenarios. The formulae of these error metrics are provided in Table 8. While RMSE emphasizes larger errors due to its squaring of residuals, MAE provides a more straightforward interpretation by averaging the absolute errors, making it less sensitive to outliers. On the other hand, sMAPE offers a symmetric perspective on percentage errors, balancing the impact of over and under forecasting. These complementary metrics help to provide a more general understanding of the forecasting performance.

**Table 8:** Normalized Error Metrics; $o_t$ is the observation (target); $f_t$ is the forecast; $\tilde{o} = \max _{t \in [1, n]} (o_t)$.

**Metrics**
$$\text {NRMSE} = \frac{100\sqrt{\frac{1}{n} \sum _{t=1}^{n} (f_t - o_t)^2}}{\tilde{o}}$$
$$\text {NMAE} = \frac{\frac{100}{n} \sum _{t=1}^{n} |f_t - o_t|}{\tilde{o}}$$
$$\text {sMAPE} = \frac{100}{n} \sum _{t=1}^{n} \frac{|f_t - o_t|}{(|f_t| + |o_t|)/2}$$

The obtained results are summarized in Table 9, where the column ‘VMD’ shows errors obtained using the 1-1 method, used as first benchmark, ’MVMD’ shows errors obtained using a the N-1 method described in^32^ and the column ‘VMDMS’ shows errors obtained using the presented method. The table cells have been color-coded with a heatmap: for each row, the best performances (lowest errors) are shown in white cells, the second-best in light red, and the highest errors in dark red cells. The first row ’P1 - Slot 1’ reports the validation set results while the other rows report the test results. The analysis of the metrics demonstrates that the proposed method pursues a performance gain over 1-1 forecasting, with a clear prevalence of lower errors. In particular, NRSME values range from a minimum of 1.59% to a maximum of 4.86%, NMAE values range from a minimum of 1.47% to a maximum of 3.70%, and sMAPE values vary within the range of 1.15%–3.58%. However, the tests show overall comparable performance when considering ’VMDMS’ and ’MVMD’, while both appear to outperform VMD.

**Table 9:**
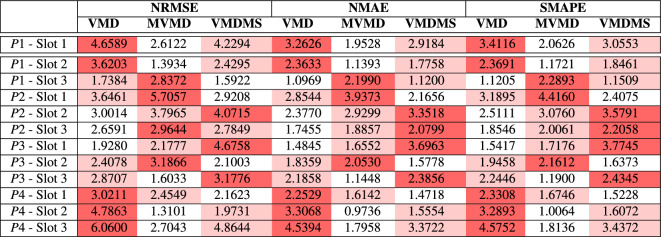
Forecasting error metrics for the proposed method and other methods with 8h forecasting horizon.

#### Analysis of error propagation in recursive forecasting

An important consideration arises when examining error propagation in iterative forecasting. While single-step forecasts can be evaluated within confidence intervals that quantify uncertainty^44^, understanding how this error evolves over time in multi-step iterative forecasting remains a complex challenge, particularly given the inherently chaotic dynamics of the iterative forecasting process. This is exemplified in Figure 9, which displays the forecasting results of the tested algorithms—VMD, MVMD, and VMDMS—corresponding to those presented in Table 9. As previously noted, all algorithms exhibit strong performance in the early forecasting steps, but their accuracy diminishes significantly afterward, reflecting a chaotic transition. This decline is likely due to the recursive nature of iterative predictions, where errors from earlier steps compound and influence subsequent forecasts.

**Fig. 10:**
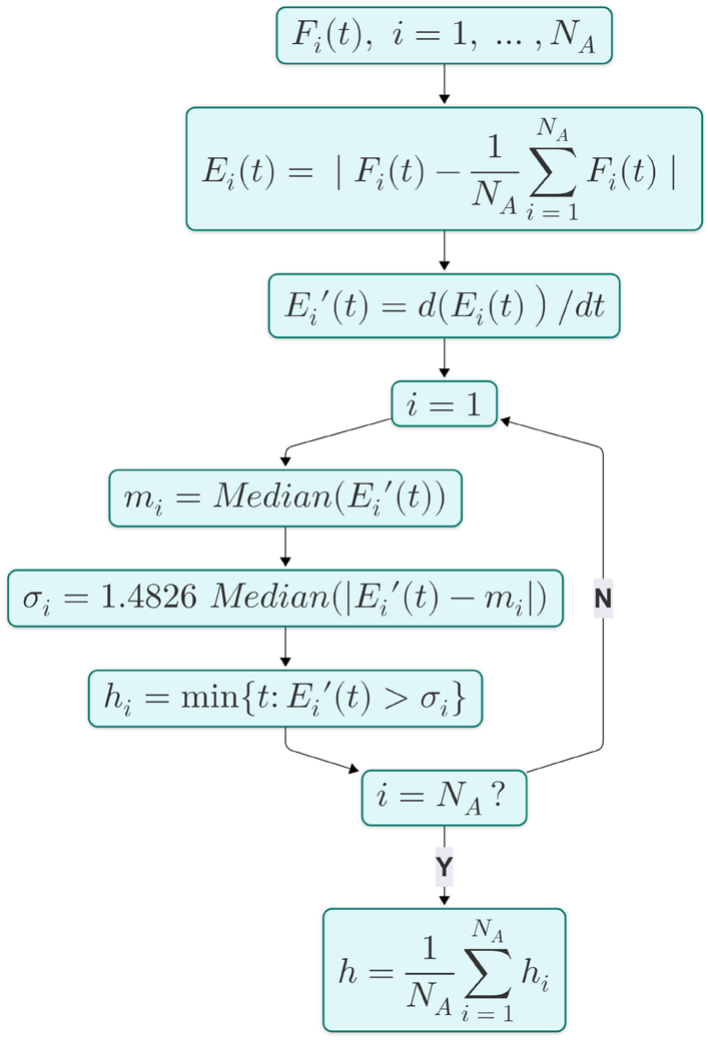
The $N_A = 3$ forecasting signals of VMD, MVMD and VMDMS, referred as $F_i(t)$ are used to compute the average forecasting horizon *h*. The process begins by computing the absolute forecast errors $E_i(t)$ across multiple algorithms. The derivative $E_i^\prime(t)$ of each error signal is then obtained to identify rapid increases. For each derivative, the median $m_i$ and the Median Absolute Deviation (MAD) are computed, with the MAD scaled by a factor of approximately 1.4826^45^. This factor converts MAD into a robust estimate of the standard deviation, serving as a threshold $\sigma _i$. The minimal time $h_i$ at which the derivative exceeds this threshold indicates a significant escalation in error, marking the predictability horizon for each algorithm. The overall horizon estimate is obtained by averaging these individual $h_i$ values.

To formalize this analysis, we employed the procedure illustrated in Figure 10 to compute a transition horizon, which delineates an initial phase, where chaotic effects are less pronounced, from a subsequent, more chaotic phase. Figure 10 illustrates the algorithm for estimating the predictability horizon based on the derivatives of forecast errors. The process begins by estimating the forecast errors $F_i(t)$ across multiple algorithms by computing the difference of each of them w.r.t. their average; then it calculates the absolute error $E_i(t)$. The derivative $E_i^\prime(t)$ of each error signal is then obtained to identify rapid increases. For each derivative, the standard deviation is estimated according to^45^ and serves as a threshold $\sigma _i$. The minimal time $h_i$ at which the derivative exceeds this threshold indicates a significant escalation in error, marking the predictability horizon for each algorithm. The overall horizon estimate is obtained by averaging these individual $h_i$ values. This procedure is repeated for each power signal and test area (a total of 12 times), resulting in a vector: [13, 15, 16, 12, 15, 11, 16, 18, 15, 17, 17, 13]. The final threshold is then obtained by taking the average of these values and conservatively reducing it by the standard deviation. The result is approximately 12.6, which corresponds to a timespan of about 2 hours.

**Table 10:**
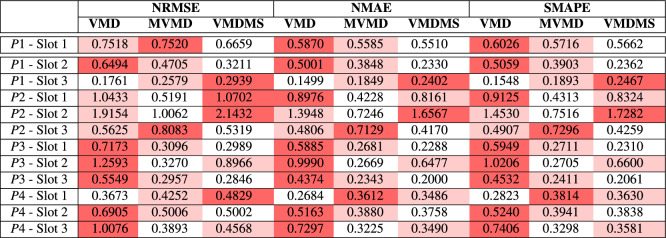
Forecasting error metrics over a 12 time step forecasting horizon.

#### Results within the 2-hour-ahead time horizon

Table 10 shows that VMDMS outperforms both benchmark methods VMD and MVMD over a two-hour forecast horizon, resulting in a more accurate solution when the forecasting is less affected by chaotic fluctuations. This result is confirmed when comparing VMDMS with the naive forecasting solutions as represented in Figure 8. Results are presented in Table 11.

**Table 11:**
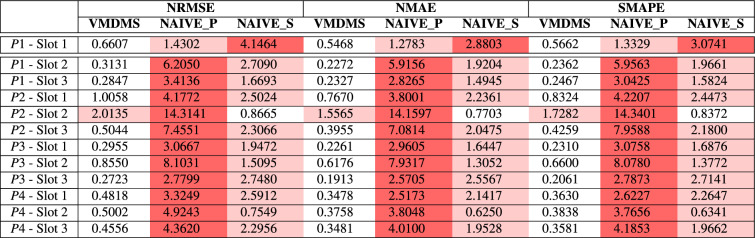
Forecasting error metrics for the proposed method VMDMS with respect to two naive solutions, Naive Persistence (NAIVE_P) and Naive Seasonal Persistence (NAIVE_S), over a 2-hour timespan.

## Conclusions

This paper addressed the topic of accurate electric load forecasting for ship energy management, a key area for optimizing efficiency in large passenger ships and aligning them with recent international decarbonization policies.

The focus of this work was to define a novel methodology for multi-step-ahead forecasting by combining a new technique for multivariate time series decomposition with an ANN architecture to achieve improved accuracy. We introduced the VMDMS algorithm, which performs multivariate input decomposition by grouping modes with similar central frequencies. This grouping allowed to leverage the most cooperative modes for the forecasting task. After decomposing the multivariate time series, the resulting components were selectively used as input for a simple yet effective forecasting model based on an LSTM and the outputs were then recomposed to generate an overall power time series prediction.

To evaluate the proposed approach, we conducted multiple tests using electric load power time series data from a large, real-world passenger ship. The results demonstrate a forecasting performance gain over univariate (1-1) forecasting across an 8-hour horizon. Notably, within the first 2 hours, where the adverse effects of the inherent chaotic behavior of iterative multistep forecasting are less pronounced, our method outperforms MVMD^32^, an alternative multivariate VMD approach.

This capability proves particularly valuable for energy managers who must optimize ship resources. They can rely on high-quality forecasting over the 8-hour horizon while simultaneously benefiting from more accurate predictions within the shorter 2-hour horizon, enabling them to fine-tune their decisions in response to potentially more stringent operational requirements.

Looking ahead to further research, while the proposed method appears to generally outperform the 1-1 forecasting case and all the other benchmarks (MVMD and the two naive approaches considered in this work) in the 2h horizon, the presence of situations where that is not the case, suggests the possibility of further refinement of the partition procedure. This refinement will include analyses of the modes’ phase shift in order to identify possible causality relationships among the time series variables.

A particularly interesting aspect concerns the interaction between multi-step iterative forecasting and its increasing influence on chaotic trends in the resulting solution. While this effect is evident in VMD and VMDMS, it appears less severe in MVMD. This is especially noticeable when comparing results over the 8-hour horizon with respect to the 2-hour horizon. The findings suggest that aligning the frequencies of cooperative modes may influence these chaotic dynamics, opening up new research avenues to investigate the relationships among these aspects.

## Electronic supplementary material

Below is the link to the electronic supplementary material.

Supplementary Material 1

## Data Availability

The data that support the findings of this study are available from Fincantieri Group, but restrictions apply to the availability of these data, which were used under license for the current study, and so are not publicly available. Data are however available from the authors upon reasonable request to the corresponding author and with permission of Fincantieri Group.

## References

[1] Dong, Y., Li, J., Liu, Z., Niu, X. & Wang, J. Ensemble wind speed forecasting system based on optimal model adaptive selection strategy: Case study in china. *Sustainable Energy Technologies and Assessments* **53**, 102535. 10.1016/j.seta.2022.102535 (2022).

[2] Dong, Y., Zhang, L., Liu, Z. & Wang, J. Integrated forecasting method for wind energy management: A case study in china. *Processes* **8**, doi: 10.3390/pr8010035(2020).

[3] Jiang, P., Liu, Z., Wang, J. & Zhang, L. Decomposition-selection-ensemble prediction system for short-term wind speed forecasting. *Electr. Power Syst. Res.* **211**, 108186. 10.1016/j.epsr.2022.108186 (2022).

[4] Oceanic, N. & (NOAA), A. A. Precipitation prediction grand challenge strategy (2020). Accessed: 2025-05-12.

[5] Vincze, M., Borcia, I. D. & Harlander, U. Temperature fluctuations in a changing climate: an ensemble-based experimental approach. *Sci. Rep.* **7**, 254. 10.1038/s41598-017-00319-0 (2017). 28325927 10.1038/s41598-017-00319-0 PMC5428220

[6] Int. Marit. Organ. Fourth imo ghg study 2020 full report. *Int. Marit. Organ.* **6**, 951–952 (2021).

[7] DNV-GL. MARITIME FORECAST TO 2050 - Energy Transition Outlook 2019. *Tech. Rep.*, DNV-GL (2019).

[8] IMO. Imo action to reduce greenhouse gas emissions from international shipping (2019).

[9] Lee, H. et al. Comparative life cycle assessments and economic analyses of alternative marine fuels: Insights for practical strategies. *Sustainability* **16**, 2114. 10.3390/su16052114 (2024).

[10] Di Piazza, M. Volume ii: Energy management systems for optimal operation of electrical micro/nanogrids. *Energies* **17**, 1811. 10.3390/en17081811 (2024).

[11] Nivolianiti, E., Karnavas, Y. L. & Charpentier, J.-F. Energy management of shipboard microgrids integrating energy storage systems: A review. *Renew. Sustain. Energy Rev.* **189, Part A**, 114012, doi: 10.1016/j.rser.2023.114012(2024).

[12] Di Piazza, M., La Tona, G., Luna, M. & Di Piazza, A. A two-stage energy management system for smart buildings reducing the impact of demand uncertainty. *Energy Build.* **139**, 1–9. 10.1016/j.enbuild.2017.01.003 (2017).

[13] Hao, X., Yin, H., Gao, J. & Lan, H. Ultra short-term forecasting for the propulsion energy consumption of all-electric ships based on tcffa-gru-parallel network. *Results in Engineering* **25**, 103767. 10.1016/j.rineng.2024.103767 (2025).

[14] Zeng, W. et al. Ultra short-term power load forecasting based on similar day clustering and ensemble empirical mode decomposition. *Energies* **16**, doi: 10.3390/en16041989(2023).

[15] Sharma, A. & Jain, S. K. A novel two-stage framework for mid-term electric load forecasting. *IEEE Trans. Industr. Inform.* **20**, 247–255. 10.1109/TII.2023.3259445 (2024).

[16] Panda, S. K., Ray, P. & Salkuti, S. R. A review on short-term load forecasting using different techniques. In Gupta, O. H., Sood, V. K. & Malik, O. P. (eds.) Recent Advances in Power Systems: Select Proceedings of EPREC-2021, 433–454, doi: 10.1007/978-981-16-6970-5_33(Springer Nature Singapore, Singapore, 2022).

[17] Wilson, G. T. Time Series Analysis: Forecasting and Control, 5th Edition , by George E. P. Box , Gwilym M. Jenkins , Gregory C. Reinsel and Greta M. Ljung , 2015 . Published by John Wiley and Sons Inc. , Hoboken, N. *J. Time. Ser. Anal.* **37**, 709–711 (2016).

[18] Kumar, S., Hussain, L., Banarjee, S. & Reza, M. Energy load forecasting using deep learning approach-lstm and gru in spark cluster. In *2018 Fifth International Conference on Emerging Applications of Information Technology (EAIT)*, 1–4, doi: 10.1109/EAIT.2018.8470406(2018).

[19] Wang, Y., Shen, Y., Mao, S., Chen, X. & Zou, H. Lasso and lstm integrated temporal model for short-term solar intensity forecasting. *IEEE Internet Things J.* **6**, 2933–2944. 10.1109/JIOT.2018.2877510 (2019).

[20] Chang, Z., Zhang, Y. & Chen, W. Electricity price prediction based on hybrid model of adam optimized lstm neural network and wavelet transform. *Energy* **187**, 115804. 10.1016/j.energy.2019.07.134 (2019).

[21] Wang, W., Shao, J. & Jumahong, H. Fuzzy inference-based lstm for long-term time series prediction. *Sci. Rep.* **13**, 20359 (2023). 37990124 10.1038/s41598-023-47812-3 PMC10663611

[22] Di Piazza, A., Di Piazza, M., La Tona, G. & Luna, M. An artificial neural network-based forecasting model of energy-related time series for electrical grid management. Mathematics and Computers in Simulation **184**, 294–305, doi: 10.1016/j.matcom.2020.05.010(2021). ELECTRIMACS 2019 ENGINEERING - Modelling and computational simulation for analysis and optimisation in electrical power engineering.

[23] Guo, Z., Zhou, K., Zhang, X. & Yang, S. A deep learning model for short-term power load and probability density forecasting. *Energy* **160**, 1186–1200. 10.1016/j.energy.2018.07.090 (2018).

[24] Rahimi, N. et al. A comprehensive review on ensemble solar power forecasting algorithms. *J. Electr. Eng. Technol.* **18**, 719–733. 10.1007/s42835-023-01378-2 (2023). 37521955 10.1007/s42835-023-01378-2 PMC9834683

[25] Liu, Y., Liang, Z. & Li, X. Enhancing short-term power load forecasting for industrial and commercial buildings: A hybrid approach using timegan, cnn, and lstm. *IEEE Open J. Ind. Electron. Soc.* **4**, 451–462. 10.1109/OJIES.2023.3319040 (2023).

[26] Dragomiretskiy, K. & Zosso, D. Variational mode decomposition. *IEEE Trans. Signal. Process.* **62**, 531–544. 10.1109/TSP.2013.2288675 (2014).

[27] Haykal, V., Cardot, H. & Ragot, N. A combination of variational mode decomposition with neural networks on household electricity consumption forecast. In P*roceedings Of ITISE 2017* (2017).

[28] Zhang, Z., Hong, W. & Li, J. Electric load forecasting by hybrid self-recurrent support vector regression model with variational mode decomposition and improved cuckoo search algorithm. *IEEE Access* **8**, 14642–14658. 10.1109/ACCESS.2020.2966452 (2020).

[29] Huang, Y., Huang, Z., Yu, J., Dai, X. & Li, Y. Short-term load forecasting based on ipso-dbilstm network with variational mode decomposition and attention mechanism. *Applied Intelligence* **53**, 12701–12718. 10.1007/s10489-022-04174-z (2023).

[30] Xiong, Q., Liu, M., Li, Y., Zheng, C. & Deng, S. Short-term load forecasting based on vmd and deep tcn-based hybrid model with self-attention mechanism. *Applied Sciences* **13**, 12479. 10.3390/app132212479 (2023).

[31] Wang, L., Zhou, X., Xu, H., Tian, T. & Tong, H. Short-term electrical load forecasting model based on multi-dimensional meteorological information spatio-temporal fusion and optimized variational mode decomposition. *IET Generation, Transmission & Distribution* **17**, 4647–4663. 10.1049/gtd2.12992 (2023).

[32] Rehman, N. & Aftab, H. Multivariate variational mode decomposition. IEEE Transactions on Signal Processing **PP**, 1–1, doi: 10.1109/TSP.2019.2951223(2019).

[33] Wang, Z., Gao, R., Wang, P. & Chen, H. A new perspective on air quality index time series forecasting: A ternary interval decomposition ensemble learning paradigm. *Technol. Forecast. Soc. Change.* **191**, 122504. 10.1016/j.techfore.2023.122504 (2023).

[34] Hao, Y., Wang, X., Wang, J. & Yang, W. A new perspective of wind speed forecasting: Multi-objective and model selection-based ensemble interval-valued wind speed forecasting system. *Energy Convers. Manag.* **299**, 117868. 10.1016/j.enconman.2023.117868 (2024).

[35] Wu, H., Liang, Y., Gao, X.-Z., Du, P. & Li, S.-P. Human-cognition-inspired deep model with its application to ocean wave height forecasting. *Expert Syst. Appl.* **230**, 120606. 10.1016/j.eswa.2023.120606 (2023).

[36] Zheng, Z. et al. Design data decomposition-based reference evapotranspiration forecasting model: A soft feature filter based deep learning driven approach. *Eng. Appl. Artif. Intell.* **121**, 105984. 10.1016/j.engappai.2023.105984 (2023).

[37] Xie, G., Jiang, F. & Zhang, C. A secondary decomposition-ensemble methodology for forecasting natural gas prices using multisource data. *Resour. Policy.* **85**, 104059. 10.1016/j.resourpol.2023.104059 (2023).

[38] Fazzini, P., La Tona, G., Diez, M. & Di Piazza, M. C. Sailing towards efficiency: A variational mode decomposition based approach to forecasting shipboard electrical power consumption. In *2024 IEEE International Conference on Electrical Systems for Aircraft, Railway, Ship Propulsion and Road Vehicles & International Transportation Electrification Conference (ESARS-ITEC)*, accepted, 1–5 (2024).

[39] Huang, N. E. et al. The empirical mode decomposition and the hilbert spectrum for nonlinear and non-stationary time series analysis. *Proc. R. Soc. Lond. A* **454**, 903–995. 10.1098/rspa.1998.0193 (1998).

[40] Eckstein, J. & Yao, W. Understanding the convergence of the alternating direction method of multipliers: Theoretical and computational perspectives. *Pacific Journal of Optimization* **11**, 619–644 (2015).

[41] Hochreiter, S. Long short-term memory. Neural Computation MIT-Press (1997). 10.1162/neco.1997.9.8.1735 9377276

[42] Sulligoi, G., Vicenzutti, A. & Menis, R. All-electric ship design: From electrical propulsion to integrated electrical and electronic power systems. *IEEE Transactions on Transportation Electrification* **2**, 507–521. 10.1109/TTE.2016.2598078 (2016).

[43] Patel, M. R. Shipboard electrical power systems (Crc Press, 2021).

[44] Ashraf, W. M. & Dua, V. Storage of weights and retrieval method (swarm) approach for neural networks hybridized with conformal prediction to construct the prediction intervals for energy system applications. *Int. J. Data. Sci. Anal.*10.1007/s41060-024-00595-w (2024).

[45] Rousseeuw, L. & Leroy, A. *Robust Regression and Outlier Detection* (Wiley, New York, 1987).

