# Supplementary material for: Enhanced forecasting of shipboard electrical power demand using multivariate input and variational mode decomposition with mode selection
Source: Sci Rep. 2025 Jul 4;15:23941. doi: 10.1038/s41598-025-06153-z (PMC12227765; doi:10.1038/s41598-025-06153-z)
Supplement: Supplementary file 1 — Supplementary Material 1 [file 41598_2025_6153_MOESM1_ESM.pdf]

Enhanced Forecasting of Shipboard Electrical Power Demand using Multivariate Input and Variational Mode Decomposition with Mode Selection

Fazzini, Paolo
paolo.fazzini@cnr.it

La Tona, Giuseppe
giuseppe.latona@cnr.it

Diez, Matteo
matteo.diez@cnr.it

Di Piazza, Maria Carmela
mariacarmela.dipiazza@cnr.it

Appendix A: VMD Development Details

The procedure ([1]) starts from the Augmented Lagrangian variational principle:

$$\begin{aligned}\mathcal{L}\left(\{u_k\},\{\omega_k\},\lambda\right) := \alpha \sum_k \left\| \partial_t \left[\left(\delta(t) + \frac{j}{\pi t} \right) * u_k(t) \right] \exp(-j\omega_k t) \right\|_2^2 \\ + \left\| f(t) - \sum_k u_k(t) \right\|_2^2 \\ + \left\langle \lambda(t), f(t) - \sum_k u_k(t) \right\rangle\end{aligned} \tag{1}$$

where α is a weighing parameter and $\lambda(t)$ is the parameter of the Lagrangian multipliers term enforcing the norm constraint. Now, making use of the Parseval/Plancherel Fourier isometry under the norm, this problem can be solved in spectral domain:

$$\hat{u}_k^{n+1} = \underset{\hat{u}_k, u_k \in X}{\arg\min} \{ \alpha \left\| j\omega \left[(1 + \operatorname{sgn}(\omega + \omega_k)) \hat{u}_k(\omega + \omega_k) \right] \right\|_2^2 + \left\| \hat{f}(\omega) - \sum_i \hat{u}_i(\omega) + \hat{\lambda}(\omega)/2 \right\|_2^2 \Bigg\} \tag{2}$$

Where for simplicity the n and $n+1$ iteration indices are omitted for the fixed directions ω_k and $u_{z,k}$ respectively, but each is implicitly understood as the most recent available update, and $X = L^1 \cap W^{2,2}$ is the definition set of f and u_k. Here L^1 denotes the space of Lebesgue integrable functions, $W^{2,2}$ denotes the Sobolev space that have square-integrable weak derivatives up to the second order, and the hat-quantities represent the Fourier-transformed versions of their corresponding variables in the time domain. Next, a change of variables $\omega \leftarrow \omega - \omega_k$ is performed:

$$\hat{u}_k^{n+1} = \underset{\hat{u}_k, u_k \in X}{\arg\min} \left\{ \alpha \left\| j(\omega - \omega_k) \left[(1 + \operatorname{sgn}(\omega)) \hat{u}_k(\omega) \right] \right\|_2^2 + \left\| \hat{f}(\omega) - \sum_i \hat{u}_i(\omega) + \hat{\lambda}(\omega)/2 \right\|_2^2 \right\} \tag{3}$$

Both terms can be re-arranged as halfspace integrals over the non-negative frequencies by leveraging the Hermitian symmetry of the real signals in the reconstruction fidelity term:

$$\hat{u}_k^{n+1} = \underset{\hat{u}_k, u_k \in X}{\arg\min} \left\{ \int_0^\infty 4\alpha (\omega - \omega_k)^2 \left| \hat{u}_k(\omega) \right|^2 + 2 \left| \hat{f}(\omega) - \sum_i \hat{u}_i(\omega) + \hat{\lambda}(\omega)/2 \right|^2 d\omega \right\} \tag{4}$$

The solution of this quadratic optimization problem is determined by letting the first variation vanish for the positive frequencies:

$$\hat{u}_k^{n+1}(\omega) = \frac{\hat{f}(\omega) - \sum_{i \neq k} \hat{u}_i(\omega) + \hat{\lambda}(\omega)/2}{1 + 2\alpha (\omega - \omega_k)^2} \tag{5}$$

which is an iterative Wiener filtering of the current residual, with prior $1/(\omega - \omega_k)^2$. A mode in time domain is obtained as the real part of the inverse Fourier transform of this filtered analytic signal. The central frequencies ω_k do not appear in the reconstruction fidelity term, but only in the bandwidth prior. The relevant problem thus reads:

$$\omega_k^{n+1} = \arg\min_{\omega_k} \left\{ \left\| \partial_t \left[\left(\delta(t) + \frac{j}{\pi t} \right) * u_k(t) \right] e^{-j\omega_k t} \right\|_2^2 \right\} \tag{6}$$

As before, the optimization can take place in Fourier domain:

$$\omega_k^{n+1} = \arg\min_{\omega_k} \left\{ \int_0^\infty (\omega - \omega_k)^2 \left| \hat{u}_k(\omega) \right|^2 d\omega \right\} \tag{7}$$

This quadratic problem is solved as:

$$\omega_k^{n+1} = \frac{\int_0^\infty \omega \left| \hat{u}_k(\omega) \right|^2 d\omega}{\int_0^\infty \left| \hat{u}_k(\omega) \right|^2 d\omega} \tag{8}$$

References

[1] Dragomiretskiy, K. & Zosso, D. Variational mode decomposition. *IEEE Transactions On Signal Processing* **62**, 531–544, DOI: 10.1109/TSP.2013.2288675 (2014).
